# National cohort study on postoperative risks after surgery for submucosal invasive colorectal cancer

**DOI:** 10.1002/bjs5.50125

**Published:** 2018-12-24

**Authors:** N. C. A. Vermeer, Y. Backes, H. S. Snijders, E. Bastiaannet, G. J. Liefers, L. M. G. Moons, C. J. H. van de Velde, K. C. M. J. Peeters, the Dutch T1 Colorectal Cancer Working Group

**Affiliations:** ^1^ Department of Surgery Leiden University Medical Centre Leiden The Netherlands; ^2^ Department of Medical Oncology, Leiden University Medical Centre Leiden The Netherlands; ^3^ Department of Gastroenterology, University Medical Centre Utrecht Utrecht The Netherlands; ^4^ Department of Hepatology, University Medical Centre Utrecht Utrecht The Netherlands; ^5^ Department of Surgery, Groene Hart Ziekenhuis Gouda The Netherlands

## Abstract

**Background:**

The decision to perform surgery for patients with T1 colorectal cancer hinges on the estimated risk of lymph node metastasis, residual tumour and risks of surgery. The aim of this observational study was to compare surgical outcomes for T1 colorectal cancer with those for more advanced colorectal cancer.

**Methods:**

This was a population‐based cohort study of patients treated surgically for pT1–3 colorectal cancer between 2009 and 2016, using data from the Dutch ColoRectal Audit. Postoperative complications (overall, surgical, severe complications and mortality) were compared using multivariable logistic regression. A risk stratification table was developed based on factors independently associated with severe complications (reintervention and/or mortality) after elective surgery.

**Results:**

Of 39 813 patients, 5170 had pT1 colorectal cancer. No statistically significant differences were observed between patients with pT1 and pT2–3 disease in the rate of severe complications (8·3 *versus* 9·5 per cent respectively; odds ratio (OR) 0·89, 95 per cent c.i. 0·80 to 1·01, *P* = 0·061), surgical complications (12·6 *versus* 13·5 per cent; OR 0·93, 0·84 to 1·02, *P* = 0·119) or mortality (1·7 *versus* 2·5 per cent; OR 0·94, 0·74 to 1·19, *P* = 0·604). Male sex, higher ASA grade, previous abdominal surgery, open approach and type of procedure were associated with a higher severe complication rate in patients with pT1 colorectal cancer.

**Conclusion:**

Elective bowel resection was associated with similar morbidity and mortality rates in patients with pT1 and those with pT2–3 colorectal carcinoma.

## Introduction

The introduction of population‐based colorectal carcinoma screening programmes aims to reduce mortality from colorectal cancer. Screening‐detected colorectal cancers have a more favourable stage distribution than those that are symptom‐detected, but it remains unclear whether early diagnosis following screening results in better surgical outcomes^1^. In January 2014, a nationwide colorectal cancer screening programme was launched in the Netherlands. Individuals aged 55–75 years are offered a biennial faecal immunochemical test (FIT), and diagnostic colonoscopy when the FIT is positive^2^.

A proportion of colorectal cancers limited to the submucosa (pT1) can be treated with minimally invasive endoscopic resection techniques, in contrast to more advanced colorectal cancers^3^. The indication to perform additional surgery depends on the risks of lymph node metastasis and incomplete resection, which are estimated using histological risk factors such as lymphovascular invasion, invasion depth, differentiation grade, tumour budding and resection margins^4^, ^5^, ^6^. Assessment of whether the oncological benefits of excision of potential positive lymph nodes and possible residual cancer tissue outweigh the risks of additional surgery is challenging^7^ ^8^. Evidence regarding the magnitude of these risks is sparse. Studies evaluating surgical morbidity and mortality of colorectal surgery consist mainly of patients with more advanced tumours^9^, ^10^, ^11^. These risks cannot simply be extrapolated to patients with pT1 colorectal cancer as the clinical characteristics of patients with advanced colorectal carcinoma might be different^12^, few treatment alternatives are available, and the risk of cancer‐related death is higher.

The aim of this study was to compare short‐term postoperative outcomes after elective bowel resection in patients with pT1 and those with pT2–3 colorectal cancer, and to identify the key clinical features associated with severe complications after surgery for pT1 colorectal cancer from which a risk stratification table could be developed to help clinicians guide treatment decisions in patients with pT1 colorectal cancer.

## Methods

This was a population‐based cohort study of patients who underwent colorectal surgery for pT1–3 stage colorectal cancer between January 2009 and December 2016 in the Netherlands. The total population in the Netherlands was estimated as 16·6 million people in 2010, according to Statistics Netherlands. Patients were identified from the Dutch ColoRectal Audit (DCRA), formerly known as the Dutch Surgical Colorectal Audit. The DCRA is a web‐based national audit, in which information on all patients undergoing surgery for a primary tumour is recorded prospectively^13^. The database has complete national coverage as the Dutch Health Inspectorate obliges inclusion of all surgically treated patients with colorectal cancer.

Patients who had an elective oncological resection were included in the study. Those who underwent neoadjuvant treatment, urgent or emergency surgery, or only a local procedure were excluded, as were patients with metastatic disease or synchronous colorectal cancer. Patients treated with a local surgical procedure before bowel resection were not excluded. As all data in the DCRA are coded, no ethical approval or informed consent was required for this study under Dutch law^14^.

### Outcomes

Main outcome measures were overall, surgical and severe complications, and mortality. Definitions are shown in *Table* 1. The reason for selecting the combined outcome of severe complications (reintervention and/or mortality) in this study was because mortality alone was considered an underestimation of the total burden to the patient^15^. If no complication was registered, the authors assumed no complication had occurred. The number of patients with surgically treated colorectal cancer was analysed over time, according to pT category, to determine the effect of the introduction of mass screening.

**Table 1:** Definitions

Definition	Description
Overall complications	Complications within 30 days after surgery including cardiac, pulmonary, thromboembolic, neurological, infectious, other general and surgical complications
Surgical complications	Complications within 30 days after primary surgery that were directly related to the surgical intervention, including anastomotic leakage, abscess, bleeding and postoperative ileus
Severe complications	Complications requiring reintervention and/or leading to death within 30 days after primary surgery (mortality)
Mortality	Death within 30 days after surgery
Reintervention	Reoperation (open or laparoscopic surgery) or radiological intervention after primary bowel surgery. Minor interventions such as placement of a central venous catheter, incision of a superficial wound infection or nasogastric intubation were not considered reinterventions

### Risk factors and study parameters

Patient‐ and tumour‐related risk factors associated with morbidity and mortality following elective colorectal surgery in previous literature were used in analyses^16^, ^17^, ^18^, ^19^. Factors analysed were: age, sex, cardiac, pulmonary and neurological co‐morbidity, ASA grade (I–II *versus* III–V), history of abdominal surgery, BMI, preoperative complications (perforation with peritonitis, abscess, obstruction or ileus, bleeding or anaemia, or other), tumour location (colon or rectum), detection method (non‐screening‐detected *versus* screening‐detected), year of surgery, type of procedure (open, laparoscopic or conversion from laparoscopic to open procedure), type of surgery (right colectomy, left colectomy, sigmoid resection, low anterior resection (LAR), abdominoperineal resection (APR), (sub)total colectomy or other), lymph node yield (less than 12 or 12 or more nodes) and pN category (N0, N1 or N2). Ileocaecal and transverse resections were also categorized as right colectomy. Panproctocolectomy and subtotal colectomy were categorized together as (sub)total colectomy. When information on co‐morbidity was missing, it was interpreted as absent. For all patients, tumour stage was defined according to the fifth edition of the TNM classification of malignant tumours for colorectal cancer^20^.

### Statistical analysis

Baseline characteristics were compared between patients with pT1, pT2 and pT3 colorectal cancer using the χ^2^ test for categorical variables and the Kruskal–Wallis test for continuous variables. Missing data were assumed to be missing at random. For all logistic regression analyses, multiple imputation using a Markov chain Monte Carlo method was performed to adjust for missing values (10‐imputation data sets, 25 iterations)^21^ ^22^.

The association between pT category (pT1 *versus* pT2–3 colorectal cancer) and short‐term postoperative outcomes was evaluated with univariable logistic regression analysis, expressed as odds ratios (ORs) with 95 per cent confidence intervals. Multivariable logistic regression analysis was performed to adjust for possible confounding factors. Age, BMI and year of surgery were analysed continuously in regression analyses; the remaining variables were analysed as categorical.

To identify risk factors associated with severe complications after elective surgery for pT1 colorectal cancer, logistic regression analyses were performed. Independent variables with *P* < 0·050 in univariable analysis were entered into the multivariable logistic regression model. A risk stratification table was developed for severe complications after surgery for pT1 colorectal cancer, stratified for sex (men *versus* women), type of operation (right colectomy *versus* left colectomy *versus* sigmoid resection *versus* LAR *versus* APR) and ASA grade (I–II *versus* III–V). Bootstrapping was performed to calculate 95 per cent confidence intervals.

GraphPad Prism® version 7.02 (GraphPad Software, La Jolla, California, USA) and Microsoft Visio® version 2010 (Microsoft, Redmond, Washington, USA) were used to draw figures. All analyses were performed in IBM SPSS® version 23.0 software (IBM, Armonk, New York, USA). Statistical significance was defined as *P* < 0·050.

## Results

**Figure 1:**
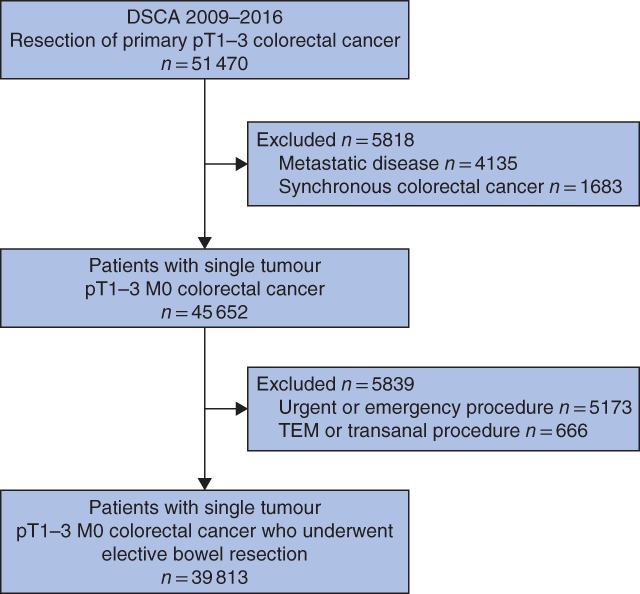
Study flow chart of included patients. DSCA, Dutch Surgical Colorectal Audit; TEM, transanal endoscopic microsurgery

Of 51 470 surgically treated patients with colorectal cancer identified, 39 813 fulfilled the inclusion criteria (*Fig*. 1). Some 5170 (13·0 per cent) were diagnosed with pT1, 9701 (24·4 per cent) with pT2 and 24 942 (62·6 per cent) with pT3 colorectal carcinoma. The mean age of the cohort was 71 years and 54·4 per cent were men. Baseline characteristics are shown in *Table* 2. Patients with T1 CRC were significantly younger, more often men, and had a lower ASA grade (all *P* < 0·001). pT1 cancers were more often screening‐detected, more frequently diagnosed in 2015–2016 and more often located in the rectum (all *P* < 0·001). Patients with pT2–3 tumours more often had preoperative complications and underwent open surgery more frequently (both *P* < 0·001). Patients treated with a local surgical procedure before bowel resection accounted for 1·3 per cent of the complete cohort. Ileocaecal and transverse resections accounted for 0·6 and 2·1 per cent of operations respectively; these were recategorized as right colectomies. Panproctocolectomy and subtotal colectomy accounted for 0·3 and 1·3 per cent respectively, and were recategorized as (sub)total colectomies.

**Table 2:** Demographic and clinical characteristics of surgically treated patients with colorectal carcinoma, according to pT category (2009–2016)

	pT1 (*n* = 5170)	pT2–3 (*n* = 34 643)	*P* ¶
Age (years)*	69(9)	71(11)	< 0·001#
Sex			< 0·001
M	2971 (57·5)	18 698 (54·0)	
F	2196 (42·5)	15 936 (46·0)	
Unknown	3 (0·1)	9 (0·0)	
Type of co‐morbidity			
Cardiac	1463 (28·3)	9924 (28·6)	0·609
Pulmonary	751 (14·5)	4851 (14·0)	0·314
Neurological	702 (13·6)	5066 (14·6)	0·047
ASA fitness grade			
I–II	4154 (80·3)	26 314 (76·0)	< 0·001
III–V	1005 (19·4)	8092 (23·4)	
Unknown	11 (0·2)	237 (0·7)	
Previous abdominal surgery			0·137
No	3300 (63·8)	22 422 (64·7)	
Yes	1857 (35·9)	12 045 (34·8)	
Unknown	13 (0·3)	176 (0·5)	
BMI (kg/m^2^)*	27(4)	27(5)	< 0·001#
Preoperative complication			< 0·001
No	4555 (88·1)	25 973 (75·0)	
Yes	584 (11·3)	8402 (24·3)	
Unknown	31 (0·6)	268 (0·8)	
Location of primary tumour			< 0·001
Colon	4397 (85·0)	31 038 (89·6)	
Rectum	773 (15·0)	3605 (10·4)	
Detection method			< 0·001
Non‐screening‐detected	3412 (66·0)	29 791 (86·0)	
Screening‐detected	1695 (32·8)	4531 (13·1)	
Unknown	63 (1·2)	321 (0·9)	
Year of surgery			< 0·001
2009–2014	2733 (52·9)	23 379 (67·5)	
2015–2016	2437 (47·1)	11 264 (32·5)	
Type of procedure			< 0·001
Laparoscopic	3784 (73·2)	20 763 (59·9)	
Laparotomy	1038 (20·1)	11 208 (32·4)	
Conversion†	327 (6·3)	2562 (7·4)	
Unknown	21 (0·4)	110 (0·3)	
Type of surgery			< 0·001
Right colectomy‡	1552 (30·0)	15 786 (45·6)	
Left colectomy	395 (7·6)	3221 (9·3)	
Sigmoid resection	2306 (44·6)	11 413 (32·9)	
LAR	644 (12·5)	2867 (8·3)	
APR	98 (1·9)	664 (1·9)	
(Sub)total colectomy§	126 (2·4)	539 (1·6)	
Other	47 (0·9)	149 (0·4)	
Unknown	2 (0·0)	4 (0·0)	
Lymph node yield			< 0·001
< 12	2229 (43·1)	7207 (20·8)	
≥ 12	2911 (56·3)	27 324 (78·9)	
Unknown	30 (0·6)	112 (0·3)	
pN category			< 0·001
pN0	4415 (85·4)	22 652 (65·4)	
pN1	496 (9·6)	8097 (23·4)	
pN2	173 (3·3)	3758 (10·8)	
Unknown	86 (1·7)	136 (0·4)	

Values in parentheses are percentages unless indicated otherwise;

* values are mean(s.d.). LAR, low anterior resection; APR, abdominoperineal resection.

† From laparoscopic to open procedure;

‡ including ileocaecal resection and transverse resection;

§ including panproctocolectomy and subtotal colectomy.

¶ χ^2^ test, except

# Kruskal–Wallis test.

### Time trends

**Figure 2:**
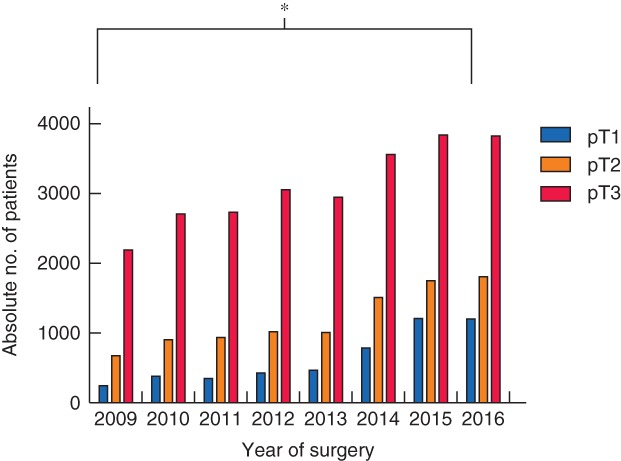
Distribution of surgically treated patients with colorectal cancer over time according to pT category. *P < 0·001 (pT1 2009 *versus* pT1 2016, χ^2^ test)

**Figure 3:**
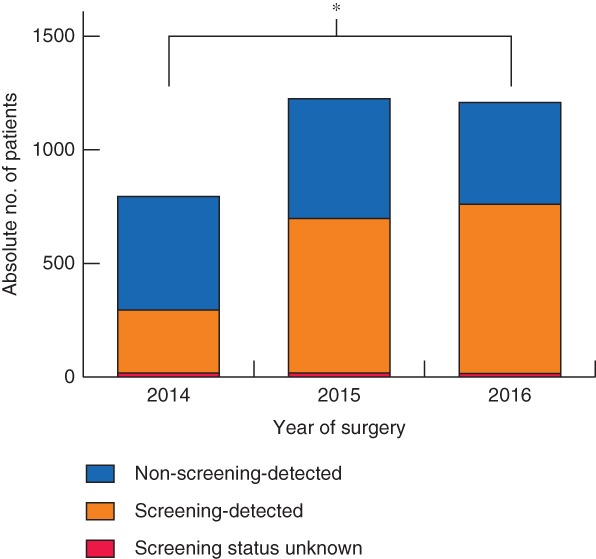
Contribution of screening‐detected tumours in patients with pT1 colorectal cancer treated surgically after implementation of mass screening programme in 2014. *P < 0·001 (χ^2^ test)

An increase in the absolute number of patients treated surgically for colorectal cancer was observed over time, from 3139 in 2009 to 6864 in 2016. The proportion of pT1 cancer increased from 8·1 per cent in 2009 to 17·7 per cent in 2016 (*P* < 0·001) (*Fig*. 2). The steepest increase was between 2014 and 2015 (+4·4 per cent), with 2014 being the year in which the colorectal cancer screening programme was introduced in the Netherlands. The proportion of screening‐detected pT1 tumours among all pT1 colorectal cancers increased from 34·6 per cent in 2014 to 61·3 per cent in 2016 (*P* < 0·001) (*Fig*. 3).

### Morbidity and mortality in pT1 *versus* pT2–3 colorectal cancer

Complications were observed in a total of 10 828 patients (27·2 per cent). Surgical complications occurred in 13·4 per cent (5334 patients) and severe complications in 9·3 per cent (3711). The 30‐day mortality rate was 2·4 per cent. The overall complication rate was significantly lower following surgery for pT1 cancer compared with surgery for pT2–3 disease (23·6 *versus* 27·7 per cent respectively; OR 0·80, 95 per cent c.i. 0·75 to 0·86, *P* < 0·001). This finding remained statistically significant after adjusting for confounders (OR 0·90, 0·84 to 0·97, *P* = 0·008). Rates of surgical complications (12·6 *versus* 13·5 per cent; adjusted OR 0·93, 0·84 to 1·02, *P* = 0·119), severe complications (8·3 *versus* 9·5 per cent; adjusted OR 0·89, 0·80 to 1·01, *P* = 0·061) and mortality (1·7 *versus* 2·5 per cent; adjusted OR 0·94, 0·74 to 1·19, *P* = 0·604) did not significantly differ between the two groups (*Table* 3). Details regarding types of complication stratified according to pT group are summarized in *Table* S1 (supporting information).

**Table 3:** Unadjusted and adjusted association between pT category of colorectal cancer (pT1 *versus* pT2–3) and postoperative outcomes

	Prevalence of outcome		Unadjusted		Adjusted‡	
	pT1 (*n* = 5170)	pT2–3 (*n* = 34 643)	Odds ratio*	*P*	Odds ratio*	*P*
Overall complications	1219 (23·6)	609 (27·7)	0·80 (0·75, 0·86)	< 0·001	0·90 (0·84, 0·97)	0·008
Surgical complications	650 (12·6)	4684 (13·5)	0·92 (0·84, 1·00)	0·062	0·93 (0·84, 1·02)	0·119
Severe complications†	427 (8·3)	3284 (9·5)	0·86 (0·77, 0·96)	0·005	0·89 (0·80, 1·01)	0·061
Mortality	87 (1·7)	880 (2·5)	0·66 (0·53, 0·82)	< 0·001	0·94 (0·74, 1·19)	0·604

Values in parentheses are percentages unless indicated otherwise;

* values in parentheses are 95 per cent confidence intervals.

† Reintervention and/or death.

‡ Adjusted for age (continuous), sex (men *versus* women), cardiac co‐morbidity, pulmonary co‐morbidity, neurological co‐morbidity, ASA grade (I–II *versus* III–V), history of abdominal surgery (yes *versus* no), BMI (continuous), preoperative complications (yes *versus* no), tumour location (rectum *versus* colon), detection method (non‐screening‐detected *versus* screening‐detected), year of surgery (continuous), type of procedure (open *versus* laparoscopic *versus* laparoscopic + conversion), type of surgery (right colectomy, left colectomy, sigmoid resection, low anterior resection, abdominoperineal resection, (sub)total colectomy or other procedure), lymph node yield (less than 12 *versus* 12 or more), pN category (N0 *versus* N1 *versus* N2).

### Risk stratification in patients with pT1 colorectal cancer

**Figure 4:**
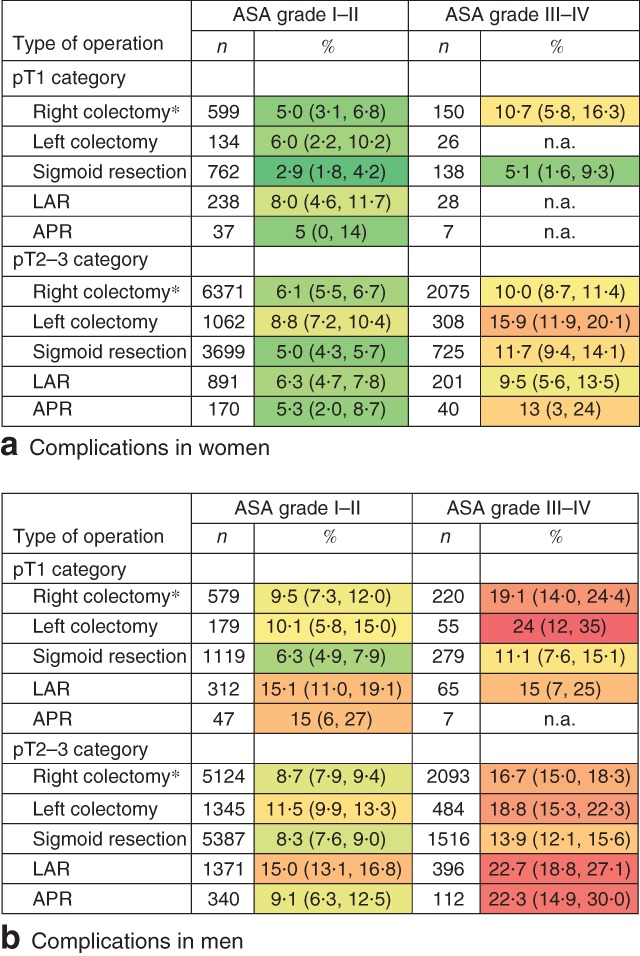
Risk of severe complications (reintervention and/or mortality within 30 days) after colorectal surgery in patients with pT1 and pT2–3 colorectal cancer. Risk of complications in **a** women and **b** men with ASA grade I–II and III–IV fitness (pT1: 427 events in 5170 patients; pT2–3: 3284 events in 34 643 patients). Increasing risk is indicated by change in colour from dark green to light green to yellow to orange to red. Values in parentheses are 95 per cent confidence intervals. *Includes ileocaecal resection and transverse resection. n.a., Not applicable (sample size too small); LAR, low anterior resection; APR, abdominoperineal resection

Factors associated with severe complications after surgery for pT1 colorectal cancer are shown in *Table* S2 (supporting information). Male sex (adjusted OR 2·21, 95 per cent c.i. 1·76 to 2·79), cardiac co‐morbidity (adjusted OR 1·26, 1·00 to 1·59), ASA grade III–IV (*versus* I–II; adjusted OR 1·41, 1·10 to 1·81), previous abdominal surgery (adjusted OR 1·25, 1·01 to 1·56), open approach (adjusted OR 1·60, 1·26 to 2·04), conversion from a laparoscopic to an open procedure (adjusted OR 1·89, 1·33 to 2·67) and subtotal colectomy (*versus* right colectomy; adjusted OR 2·38, 1·40 to 4·05) were independently associated with an increased risk of severe complications. Sigmoid resection was associated with a lower risk of severe complications (*versus* right colectomy; adjusted OR 0·67, 0·52 to 0·87). Using these risk factors, severe complication risk was stratified (*Fig*. 4). Women with ASA grade I–II and pT1 disease who underwent right colectomy or sigmoid resection had the lowest risk of severe complications (5 per cent or less), whereas men with ASA grade III–IV and pT1 disease treated with right or left colectomy had the highest risk of severe complications (more than 19 per cent).

Severe complication risks of surgery for pT2–3 colorectal cancer stratified for the same risk factors showed similar results. Women with ASA grade I–II who underwent sigmoid resection had a 5 per cent risk of severe complications and men with ASA grade III–IV treated with left colectomy had an 18·8 per cent risk (*Fig*.  4).

## Discussion

This population‐based cohort study demonstrates that patients undergoing elective bowel resections for pT1 colorectal cancer have similar risks for surgical complications, severe complications and mortality as those undergoing elective bowel resections for pT2–3 colorectal carcinoma. The absolute difference in overall complication rate following pT1 *versus* pT2–3 resection was, although statistically significant, considered minor and therefore of little clinical relevance. Implementation of colorectal cancer screening aims to increase cancer‐specific survival by diagnosing disease at an earlier stage, but also introduces treatment dilemmas. Early‐stage tumours do not necessarily lead to safer surgical procedures.

The risks of postoperative complications after elective surgery for pT1 colorectal cancer have not been well described in previous studies. This is surprising because this type of surgery is frequently performed in clinical practice. Existing literature has focused mainly on advanced stage tumours in patients undergoing emergency surgery, and includes limited analysis of mortality with no morbidity estimates. In the present study an overall postoperative 30‐day mortality rate of 2·4 per cent was observed for all patients, comparable with previous population‐based studies^11^ ^12^, ^23^, ^24^, ^25^ evaluating mortality risk in patients undergoing elective colorectal cancer resection (1·8–3·5 per cent). Previous reported relaparotomy rates after surgery for colorectal cancer range from 5·8 to 7·2 per cent^26^, in accordance with the present study. A recently published study^27^ on surgical risks after surgery for non‐malignant colorectal polyps showed a low overall 30‐day mortality rate of 0·7 per cent and a postoperative adverse event rate of 14 per cent. This, however, might be an underestimation as the American College of Surgeons' National Surgical Quality Improvement Program is not representative of all hospitals in the USA. A recently published multicentre study^28^ from the Netherlands with more than 900 patients undergoing surgery for benign colorectal polyps showed a 30‐day mortality rate of 1·4 per cent, which is more in line with the present findings.

Risk factors for severe complications after pT1 colorectal cancer surgery included sex, ASA grade, previous abdominal surgery, type of procedure and type of surgery. This is in line with previous publications, as these factors are frequently used in prognostic scoring for colorectal cancer surgery^16^ ^18^, ^19^ ^29^, ^30^. Most of these existing scoring systems have been based on data of patients with more advanced colorectal carcinoma and include factors such as urgency, perioperative contamination, disseminated cancer, ascites and signs of hypovolaemic shock, which are irrelevant in most early‐stage colorectal cancers^29^. The predictive model of the Association of Coloproctology of Great Britain and Ireland was based on a cohort in which 90 per cent of patients had advanced colorectal cancer^31^. The data used to produce the colorectal (CR)‐POSSUM model were taken from a wide range of procedures, and more than 30 per cent of the 6790 included procedures were non‐elective^32^. In the present study, patient factors such as age, co‐morbidity, BMI, tumour location, screening status and pN status were not predictive for severe complications. There has been long‐standing controversy about whether age and higher BMI are associated with worse perioperative outcomes. A recent meta‐analysis^10^ of the effect of BMI failed to show significant influence on overall mortality or reoperation/reintervention rate after laparoscopic colorectal surgery.

A major strength of this study is its nationwide population‐based design. Data are compared annually with those in the National Cancer Registry, and show nearly 100 per cent completeness^13^ ^14^, thereby reflecting daily clinical practice. It should be emphasized that patients who had neoadjuvant treatment or were operated on in the emergency setting were not included to avoid major confounding of postoperative outcomes. Several limitations should be mentioned. Inherent to a retrospective analysis, unmeasured confounding could be a source of bias. Although adjustment was made for possible confounders in multivariable analyses including screening status, a healthy user bias cannot be excluded. In previous papers, common factors such as educational level and regular check‐up experience were identified as determinants of participation in colorectal cancer screening^33^. Therefore, screened participants could be less vulnerable for postoperative complications, regardless of pT status. The stratified risk model might slightly overestimate the actual risk, because of the decline of short‐term mortality after colorectal surgery in the past decade, which was shown in this study as well as in other population‐based studies^24^. Finally, the proportion of patients with pT1 colorectal cancer that was clinically staged correctly was not known. Diagnosis by endoscopy or imaging can be misleading and either overestimate or underestimate the actual tumour stage. This may influence surgical risks and oncological benefit in either direction.

Screening programmes target a population regardless of life expectancy. Additional surgery in patients with high‐risk pT1 colorectal cancer should be well considered. Clinicians should estimate the patient's competing risks of morbidity and mortality. The risk stratification (*Fig*.  4) helps to estimate individual risks of significant morbidity and can be used before surgery in shared decision‐making of whether or not to perform completion surgery for pT1 colorectal cancer.

## Disclosure

The authors declare no conflict of interest.

## Supporting information


**Table S1** Short‐term outcomes (within 30 days) of surgically treated patients with colorectal carcinoma (2009–2016)
**Table S2** Univariable and multivariable analyses of variables associated with severe complication rate following colorectal surgery for pT1 colorectal cancer
