# Supplementary material for: National cohort study on postoperative risks after surgery for submucosal invasive colorectal cancer
Source: BJS Open. 2018 Dec 24;3(2):210–7. doi: 10.1002/bjs5.50125 (PMC6433330; doi:10.1002/bjs5.50125)
Supplement: Supplementary file 1 — Table S1 Short‐term outcomes (within 30 days) of surgically treated patients with colorectal carcinoma (2009–2016) Table S2 Univariable and multivariable analyses of variables associated with severe complication rate following colorectal surgery for pT1 colorectal cancer [file BJS5-3-210-s001.docx]

**BJS5_50125**

**National cohort study on postoperative risks after surgery for submucosal invasive colorectal cancer**

**N. C. A. Vermeer, Y. Backes, H. S. Snijders, E. Bastiaannet, G. J. Liefers, L. M. G. Moons, C. J. H. van de Velde and K. C. M. J. Peeters**

**Table S1 Short-term outcomes (within 30 days) of surgically treated patients with colorectal carcinoma (2009–2016)**

|  | **T1**  ***n*=5 170** | **T2-3**  ***n*=34 643** | ***P*-value** |
| --- | --- | --- | --- |
|  | *n* (%) | *n* (%) |  |
| Overall complications  No  Yes  Unknown | 3 942 (76.2)  1 219 (23.6)  9 (<1) | 24 916 (71.9)  9 606 (27.7)  118 (0.3) | <0.001 |
| Surgical complications  No  Yes | 4 520 (87.4)  650 (12.6) | 29 959 (86.5)  4 684 (13.5) | 0.064 |
| Other complications  Pulmonary  Cardiac  Thromboembolic  Infection (other than pulmonary/surgical)  Neurological  Other | 185 (3.6)  109 (2.1)  23 (<1)  124 (2.4)  54 (1.0)  289 (5.6) | 1 610 (4.6)  1 057 (3.1)  188 (0.5)  1 160 (3.3)  435 (1.3)  1 892 (5.5) | 0.001  <0.001  0.409  <0.001  0.224  0.697 |
| Mortality  No  Yes  Unknown | 5 074 (98.1)  87 (1.7)  10 (<1) | 33 618 (97.0)  880 (2.5)  145 (0.4) | 0.002 |
| Cause of death^  CRC  Surgery  Other cause  Unknown | 0  38 (44)  32 (37)  17 (20) | 9 (1)  333 (37.8)  327 (37.2)  211 (24.0) | 0.521 |
| Anastomotic leakage* | 176 (3.7) | 1 247 (3.9) | 0.358 |
| Re-intervention | 369 (7.1) | 2 645 (7.6) | 0.216 |
| Re-admission | 254 (4.9) | 1 706 (4.9) | 0.997 |
| Severe complications (re-intervention and/or mortality) | 427 (8.3) | 3 284 (9.5) | <0.001 |

*^ Percentage of patients deceased<30d, * percentage of patients with anastomosis*

**Table S2 Univariable and multivariable analyses of variables associated with severe**

**complication rate following colorectal surgery for pT1 colorectal cancer**

| **Parameter** | **Univariable analyses** | | | **Multivariable analyses** | | |
| --- | --- | --- | --- | --- | --- | --- |
|  | OR | CI | P-value | OR | CI | P-value |
| **Age, years** | **1.02** | **1.01-1.04** | **<0.001** | 1.01 | 1.00-1.02 | 0.062 |
| **Gender** |  |  |  |  |  |  |
| Female | Reference |  |  | Reference |  |  |
| Male | **2.15** | **1.72-2.69** | **<0.001** | **2.21** | **1.76-2.79** | **<0.001** |
| **Comorbidity** |  |  |  |  |  |  |
| Cardiac | **1.71** | **1.39-2.10** | **<0.001** | **1.26** | **1.00-1.59** | **0.049** |
| Pulmonary | **1.57** | **1.23-2.02** | **<0.001** | 1.28 | 0.99-1.67 | 0.064 |
| Neurological | 0.94 | 0.70-1.26 | 0.660 |  |  |  |
| **ASA** |  |  |  |  |  |  |
| I-II | Reference |  |  | Reference |  |  |
| III-V | **1.95** | **1.57-2.43** | **<0.001** | **1.41** | **1.10-1.81** | **0.007** |
| **Previous abdominal surgery** |  |  |  |  |  |  |
| No | Reference |  |  | Reference |  |  |
| Yes | **1.27** | **1.04-1.56** | **0.019** | **1.25** | **1.01-1.56** | **0.041** |
| **BMI** | 1.02 | 1.00-1.05 | 0.051 |  |  |  |
| **Preoperative complication** |  |  |  |  |  |  |
| No | Reference |  |  |  |  |  |
| Yes | 1.29 | 0.96-1.72 | 0.088 |  |  |  |
| **Tumour location** |  |  |  |  |  |  |
| Colon | Reference |  |  | Reference |  |  |
| Rectum | **1.61** | **1.26-2.06** | **<0.001** | 0.87 | 0.25-2.99 | 0.824 |
| **Detection method** |  |  |  |  |  |  |
| Non-screen detected | Reference |  |  |  |  |  |
| Screen-detected | 0.84 | 0.67-1.04 | 0.112 |  |  |  |
| **Year of surgery** | 1.05 | 1.00-1.10 | 0.058 |  |  |  |
| **Type of procedure** |  |  |  |  |  |  |
| Laparoscopic | Reference |  |  | Reference |  |  |
| Open | **1.91** | **1.52-2.40** | **<0.001** | **1.60** | **1.26-2.04** | **<0.001** |
| Laparoscopic + conversion^^^ | **2.34** | **1.68-3.27** | **<0.001** | **1.89** | **1.33-2.67** | **<0.001** |
| **Type of surgery** |  |  |  |  |  |  |
| Right colectomy* | Reference |  |  | Reference |  |  |
| Left colectomy | 1.11 | 0.77-1.61 | 0.579 | 1.10 | 0.76-1.61 | 0.611 |
| Sigmoid resection | **0.59** | **0.46-0.76** | **<0.001** | **0.67** | **0.52-0.87** | **0.002** |
| LAR | 1.34 | 1.00-1.80 | 0.052 | 1.86 | 0.52-6.60 | 0.341 |
| APR | 1.25 | 0.65-2.39 | 0.508 | 1.68 | 0.42-6.63 | 0.462 |
| (Sub)total colectomy^#^ | **2.08** | **1.28-3.41** | **0.003** | **2.38** | **1.40-4.05** | **0.001** |
| Other | 0.64 | 0.20-2.09 | 0.463 | 0.62 | 0.18-2.18 | 0.454 |
| **Lymph node yield** |  |  |  |  |  |  |
| <12 | Reference |  |  |  |  |  |
| >12 | 1.12 | 0.92-1.38 | 0.255 |  |  |  |
| **Pathological N-stage** |  |  |  |  |  |  |
| N0 | Reference |  |  |  |  |  |
| N1 | 0.76 | 0.52-1.09 | 0.134 |  |  |  |
| N2 | 0.58 | 0.30-1.15 | 0.117 |  |  |  |

*OR = Odds Ratio, 95% CI = 95% Confidence Interval LAR = Low Anterior Resection, APR = Abdominoperineal resection*

*^^^ From laparoscopic to open procedure, * Including ileocecal resection and transverse resection, ^#^ Including panproctocolectomy and subtotal colectomy*
